# Maslinic acid improves quality of life by alleviating joint knee pain in the elderly: results from a community-based pilot study

**DOI:** 10.3164/jcbn.16-119

**Published:** 2017-05-16

**Authors:** Satoshi Fukumitsu, Tetsu Kinoshita, Myra O. Villareal, Kazuhiko Aida, Akihiro Hino, Hiroko Isoda

**Affiliations:** 1 Nippon Flour Mills Co., Ltd., Innovation Center, 5-1-3 Midorigaoka, Atsugi, Kanagawa 243-0041, Japan; 2 Alliance for Research on North Africa (ARENA), University of Tsukuba, 1-1-1 Tennodai, Tsukuba, Ibaraki 305-8572, Japan; 3 Institute of Community Life Sciences Co., Ltd., Social Epidemiology Institute, Matsuyama, Ehime 791-1102, Japan; 4 Faculty of Life and Environmental Sciences, University of Tsukuba, 1-1-1 Tennodai, Tsukuba, Ibaraki 305-8572, Japan

**Keywords:** olive fruit extract, maslinic acid, physical quality of life, chronic knee joint pain

## Abstract

Chronic knee joint pain is common in the elderly and associated with poor quality of life. This study, an open-label clinical trial, aimed to examine how the intake on a daily basis of maslinic acid-containing product (30 mg maslinic acid) on 29 elderly residents (mean 70.7 ± 10.1 years) of Nakajima Island, Ehime, Japan. Study participants consumed 10 g jelly containing maslinic acid daily for 16 weeks and at 0 (baseline), 4, 8, 12 and 16 weeks, assessed for health-related quality of life (Short Form-8) and knee pain score (Japanese Knee Osteoarthritis Measure). After 16 weeks, the physical quality of life, more specifically, the level of Bodily Pain and Physical Component Summary, but not mental quality of life, was significantly improved by maslinic acid intake. Furthermore, maslinic acid intake significantly decreased the Japanese Knee Osteoarthritis Measure at week 8 and tended to decrease Visual Analogue Scale score at weeks 4 and 16. These results suggest that consumption of maslinic acid has a protective effect against chronic knee pain in elderly residents in a community where knee pain causes high quality of life burden.

## Introduction

Japan is now facing the advent of super-aged society more rapidly than any other country in the world. In 2014, population of aged 65 years and over accounted for 26% of the Japanese population.^(1)^ With this increase in the number of elderly people, more people are becoming concerned about health problems associated with aging. The Japanese Orthopaedic Association (JOA) proposed the “locomotive syndrome” concept to identify, among middle-aged and elderly individuals, those who are at high risk of requiring health care services because of problems with locomotion^(2)^ which is defined as having difficulty in the ability to walk or lead a normal life owing to a dysfunction in one part or more of the locomotive system such as cartilages, bones, and muscles.^(2)^

The Nakajima Island in the Kutsuna Islands is famous as a major producer of citrus fruit in west Japan. But now, Nakajima Island population has more people aged 65 years or older compared to the national average because of the shrinking young population. The population-aging rate, over the age of 65, of Nakajima Island population is over 50%, reflecting the aging rate of Japan in the next 50 years.^(3)^ Thus, we consider that the aging population inevitably will have a great impact on social systems, including public health.

**Fig. 1:**
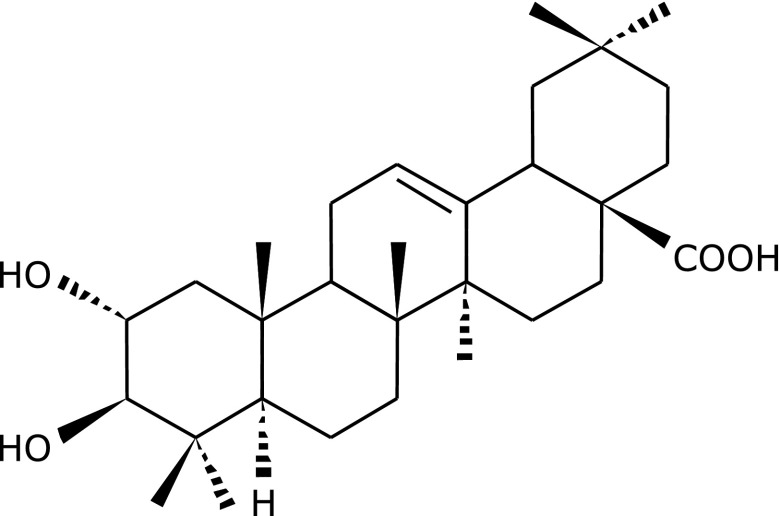
Chemical structure of maslinic acid (2α,3β-dihydroxyolean-12-en-28-oic acid).

It is a well-accepted fact that the Mediterranean diet, with its high olive oil, fiber, fruit, vegetables, and fish content, and traditionally tied to areas that grow olives, has beneficial effects on health.^(4–6)^ It has been reported that adult life expectancy for populations in the Mediterranean region is among the highest in the world, and with the incidence of other diet-related chronic diseases as among the lowest in the world in the early 1960s.^(4)^ One of the components of the Mediterranean diet, olive fruit is consumed as table olive or pressed for olive oil and has been widely accepted in Japan. In our previous study, we identified maslinic acid (MA) (2α,3β-dihydroxyolean-12-en-28-oic acid) (Fig. 1) as one of the main active components of olive fruit, in addition to the pentacyclic triterpenes such as oleanolic acid, uvaol and erythrodiol,^(7)^ and obtained evidence, *in vitro* and *in vivo*, that MA is an effective anti-inflammation and anti-arthritis component of olives.^(8–10)^ In addition, we demonstrated that MA in olive fruit is likely to improve joint pain and physical components of QOL by promoting weight loss and having an anti-inflammation effect in humans with mild knee joint pain.^(11)^ Therefore, MA derived from olive fruit product has potential as a food fortification for locomotive syndrome in today’s aging Japanese society and will likely contribute to a higher adult healthy life expectancy.

In the present study, a ready-to-eat jelly product containing MA in olive fruit extract was prepared, and given to elderly volunteers daily. Then, we conducted a preliminary research on the elderly with chronic knee pain who are residing in an isolated island (Nakajima of Ehime in Japan) to investigate whether MA-containing product intake is can be an effective way to manage the health of the elderly in order to prevent falls and promote QOL improvement in a super-aged society in the future.

## Materials and Methods

### Participants

A total of 35 subjects with a mean age of 70.7 ± 10.1 years were drawn from recruited participants in Nakajima Island (Ehime, Japan) from May to November 2015. Ethical approval was obtained from the Ethical Committee of Ehime University Medical School-Affiliated Hospital in accordance with the Declaration of Helsinki. All the 35 participants gave written informed consent at the time of enrolment, however six dropped out leaving us with a total of 29 participants data for analysis. Recruited subjects hailed from Miyano area (*n* = 13) and Oura area (*n* = 16). This clinical study is a preliminary study and was not registered in a publicly accessible database.

The inclusion criteria were: (1) male or female participants reside within Nakajima Island (Ehime, Japan), (2) at least 40 years old, (3) suffering from chronic knee pain and (4) capable of understanding and signing an informed consent form.

Exclusion criteria were: (1) with history of food allergies, (2) pregnant and lactating, and (3) other criteria rendering the participant as “inadequate” by the head of research.

### Test product for improvement of joint support

 Subjects consumed either 30 mg of MA as 300 mg as olive fruit extract in jelly once a day. Every day, each subject recorded whether (or not) he/she took the jelly during breakfast, their general physical condition, and the degree of knee pain. The staff checked the records once a month and gave the participants comments that will help motivate them in following the required schedule of taking the supplement and recording the relevant data.

Analysis of the nutritional content of olive fruit extract revealed that it contains 10.4% MA, 62.1% carbohydrate, 0.4% protein, 33.5% fat, 1.5% ash, 2.5% moisture, and other minor components such as oleanolic acid. The composition of test jellies is listed in Table 1. The MA content of the test sample was determined using HPLC as described previously.^(11)^ Briefly, HPLC separation was performed using Shimadzu Prominence HPLC system (Shimadzu Corporation, Kyoto, Japan) equipped with Inertsil ODS-3 column (150 mm × 4.6 mm, 5.6 µm; GL sciences, Inc. Tokyo, Japan). The mobile phase consisted of acetonitrile/methanol/water/phosphoric acid (500:400:100:0.5; v/v/v/v) at a flow rate of 1 ml/min for 15 min. The column was set at 30°C. Typical retention time of MA is about 4.8 min.

### Study design

**Fig. 2:**
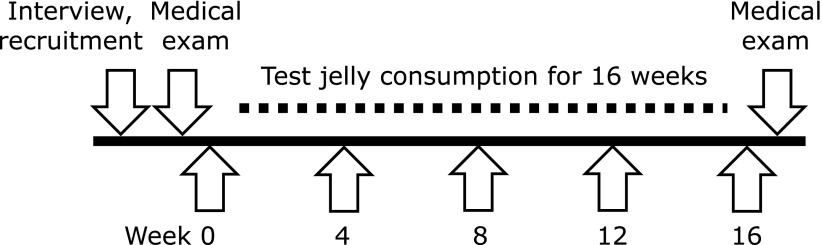
Study design for the 16-week study. Arrows indicate visits to the community center, assessment of visual analogue scale and Short Form 8 at baseline and after 4, 8, 12 and 16 weeks. Assessment of Japanese Knee Osteoarthritis Measure at baseline and after 8 and 16 weeks.

This study was conducted as an open-label study, so no placebo control was used. Each participant consumed one jelly containing 30 mg MA every day for 16 weeks. Fig. 2 summarizes the study design. Participants were supervised by the research leader to ensure that there will be no changes in the participants’ lifestyles during the study.

### Outcome assessment

The main recorded parameters were a visual analogue scale (VAS)^(12)^ to measure the level of a person’s subjective perception of pain (from 0 = no pain to 100 = maximum pain) and a widely used and well-accepted research tool, Japanese Knee Osteoarthritis Measure (JKOM)^(13)^ for Japanese patients with knee osteoarthritis, and the Short Form-8 Healthy Survey (SF-8)^(14)^ questionnaire to measure the general aspect of health-related quality of life (QOL). In brief, The JKOM is a patient-based, self-answered evaluation score that includes 25 items in 4 subcategories (pain and stiffness, activities of daily living, social activities, and general health condition), with 100 points as the maximum score. The JKOM is higher in patients with more painful physical disabilities. SF-8 measures the eight health domain consisting of physical functioning (PF), role physical (RP), bodily pain (BP), general health (GH), vitality (VT), social functioning (SF), role emotional (RE), and mental health (MH). The “Manual of the SF-8 Japanese Version” was used in this study.^(15)^ This survey provides psychometrically-based physical component summary (PCS) and mental component summary (MCS) scores. The mean score for the Japanese general population is 50 points for each domain and summary. A score of <50 was considered to indicate impaired health-related QOL, and higher scores indicated a good level of functioning and well-being in comparison with the Japanese general population.^(15)^ VAS and SF-8 were performed before and after 4, 8, 12 and 16 weeks. JKOM was performed at before and after the 8-week and 16-week period. Secondary outcome measures were obtained at 0, 4, 8, 12 and 16 weeks using body composition.

### Baseline characteristics

We recorded the subject’s age, gender, body weight, body mass index (BMI), VAS for pain, JKOM for measuring of physical function and SF-8 for health-related QOL questionnaire, and considered them as the baseline characteristics of the study subjects in the Miyano area (*n* = 13), Oura area (*n* = 16) and total in Nakajima Island (Table 2).

### Safety

Information on all the adverse events, any adverse clinical symptoms, syndrome, or illness that occurs or worsens were collected. In addition, abnormal value and worsened value for medical examination of clinical studies were considered adverse events. All the data on adverse events that occurred during the study were collected after test supplementation.

### Statistical analyses

Analysis of the participants’ data in the completed was performed and was expressed as mean ± SD. The intent-to-treat analysis was performed using SAS 9.4 for windows. The *p* value of the change in each indicator was considered significant at *p* value of 0.05. Statistical analysis was used to compare data of subjects within a group (before and after treatment), and was performed using Wilcoxon signed-rank test, a non-parametric test for independent samples.

## Results

### Baseline characteristics and compliance with the protocol

Thirty-five subjects participated until the study completion. But, six subjects discontinued their participation in the study when they felt unwell or disliked the taste of test products, leaving 29 subjects whose data were included for further analysis. All subjects maintained their usual diet and lifestyle patterns during the 16-week study. The participants’ baseline characteristics, which include the subject’s age, VAS for pain, JKOM score for measuring of physical function, and SF-8 for health-related QOL questionnaire (PF, BP, RE, MH, and PCS) between Miyano and Oura area at baseline, were statistically significant as shown in Table 2. The subjects from Miyano are significantly older than Oura (Miyano: 73.6 ± 13.6, Oura: 68.4 ± 6.3, *p* = 0.01) (Table 2). Baseline of VAS for pain and JKOM score for physical disabilities at Miyano’s subjects are significantly higher than those of Oura’s [VAS: 41.3 ± 19.0 vs 19.2 ± 28.6 (*p*<0.01), JKOM score: 28.5 ± 13.6 vs 18.6 ± 21.1 (*p*<0.01), respectively] (Table 2). Baseline of PF, BP, RE, MH, and PCS at Miyano’s subjects are significantly lower than subjects from Oura [PF: 41.6 ± 4.3 vs 46.0 ± 10.1 (*p* = 0.01), BP: 42.5 ± 8.2 vs 48.7 ± 7.8 (*p* = 0.04), RE: .43.7 ± 5.9 vs 49.7 ± 5.8 (*p*<0.01), MH: 46.6 ± 5.2 vs 51.2 ± 6.3 (*p* = 0.03), PCS: 41.1 ± 6.3 vs 44.5 ± 11.3 (*p* = 0.03)] (Table 2).

### Major examination findings

**Fig. 3:**
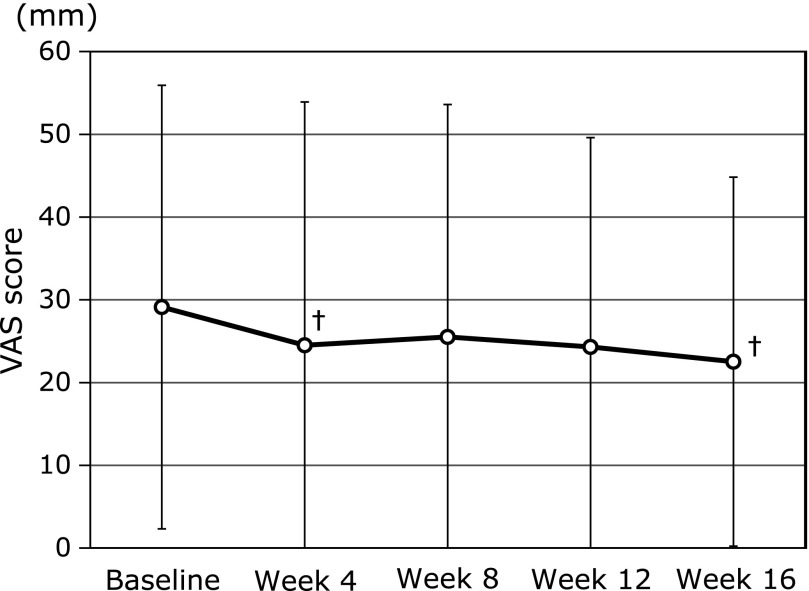
Visual analogue scale joint pain scores at baseline and after 4, 8, 12 and 16 weeks of daily ingestion of maslinic acid jelly. The values in the graph represent mean ± SD. ^†^*p*<0.1 vs baseline.

**Fig. 4:**
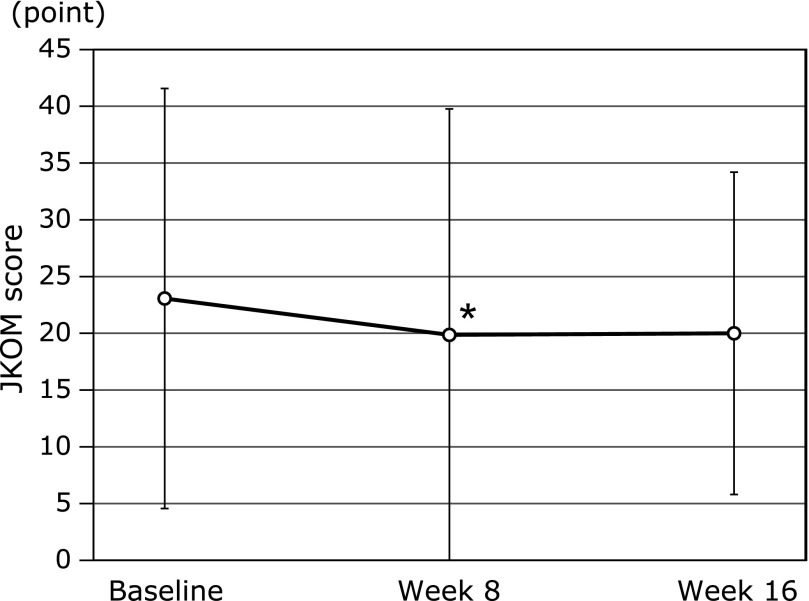
Japanese Knee Osteoarthritis Measure score at baseline, 8 and 16 weeks of daily ingestion of maslinic acid jelly. The values in the graph represent mean ± SD. *p<0.01 vs baseline.

VAS score and JKOM are shown in Fig. 3 and 4. As shown in Fig. 3, VAS score tend to decrease from baseline to 4 and 16 weeks after MA jelly consumption (29.1 ± 26.8 to 24.5 ± 29.4 points and 29.1 ± 26.8 to 22.5 ± 22.3 points, *p* = 0.06 and 0.08, respectively). In addition, as shown in Fig. 4, JKOM score was significantly reduced by 8 weeks of MA jelly consumption compared to baseline (23.1 ± 18.5 to 19.9 ± 19.9 points, *p* = 0.04) although there was no significant differences between the baseline and week 16 (23.1 ± 18.5 to 20 ± 14.2 points, *p* = 0.13).

**Fig. 5:**
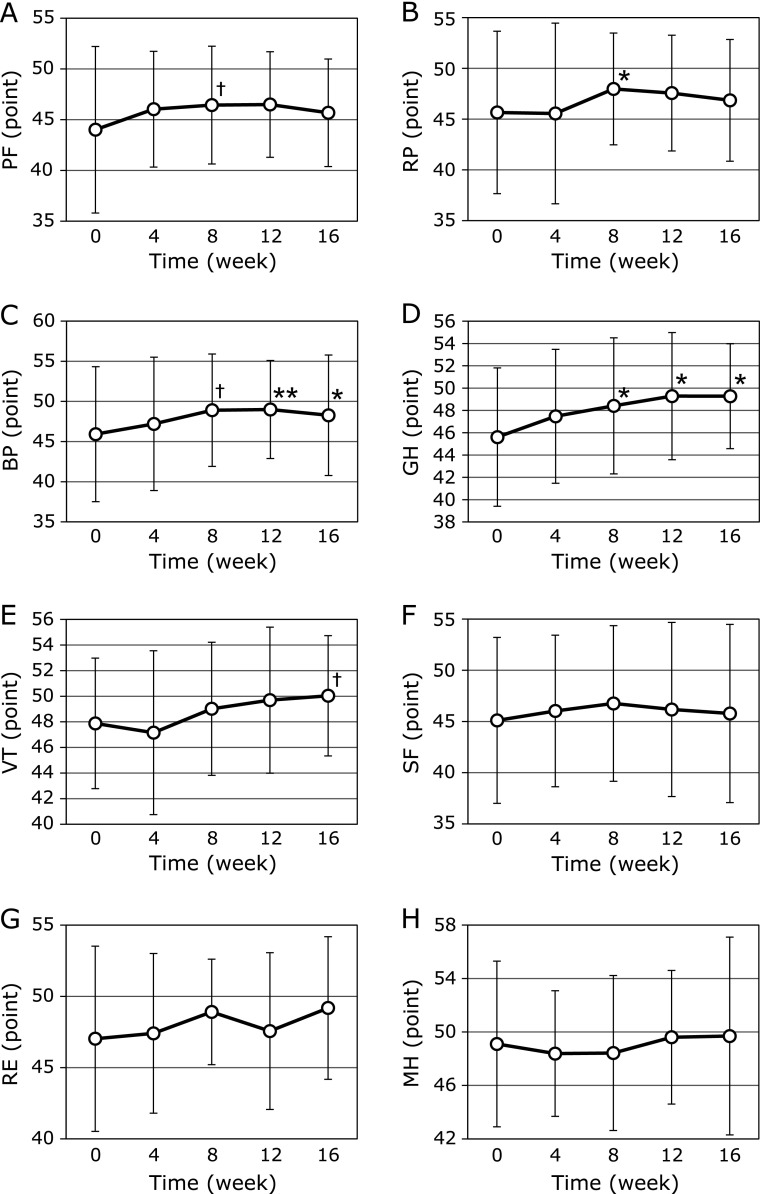
Short Form 8 scores at baseline, 4, 8, 12 and 16 weeks of daily ingestion of maslinic acid jelly. The values in the graph represent mean ± SD. ^†^*p*<0.1, *p<0.05, **p<0.01 vs baseline. Physical function (PF), role physical (RP), bodily pain (BP), general health (GH), vitality (VT), social functioning (SF), role emotional (RE), mental health (MH).

**Fig. 6:**
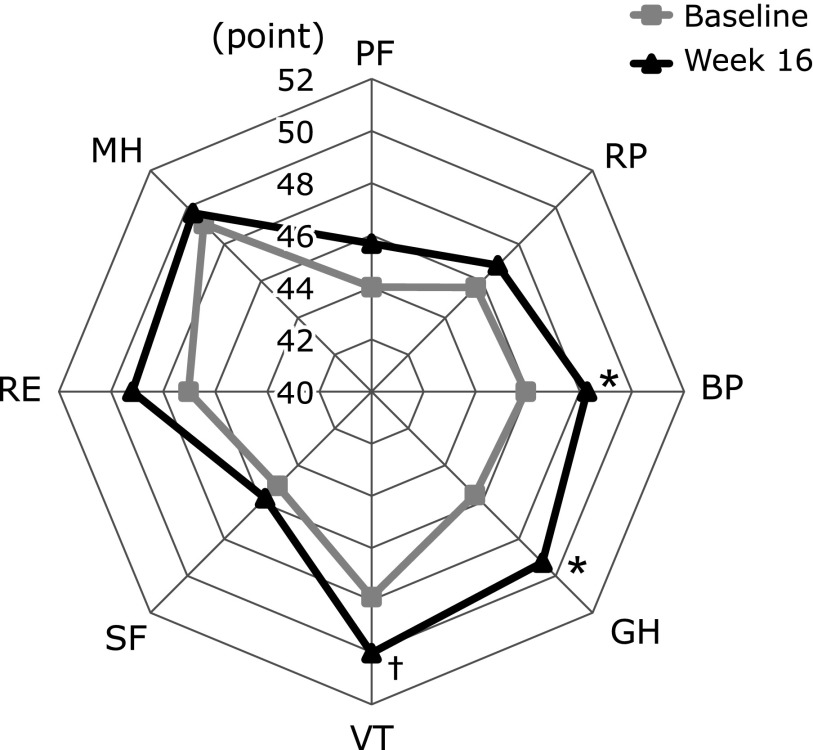
Average Short Form 8 scores at baseline and 16 weeks on the effect of maslinic acid intake on the quality of life (QOL) parameters: physical function (PF), role physical (RP), bodily pain (BP), general health (GH), vitality (VT), social functioning (SF), role emotional (RE), mental health (MH). ^†^*p*<0.1, *p<0.05 vs baseline.

**Fig. 7:**
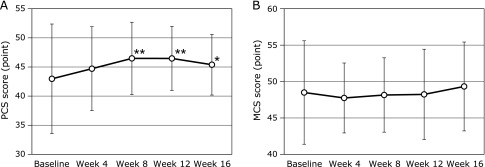
Short Form 8 summary scores at baseline, 4, 8, 12 and 16 week of daily ingestion of maslinic acid jelly. The values in the graph represent mean ± SD. *p<0.01 vs baseline. PCS, physical component summary; MCS, mental component summary.

This study also observed that participants experienced a better quality of life (SF-8) after taking the MA product at 4, 8, 12 and 16 weeks compared to baseline (Fig. 5). For the changes before and 16 weeks the intervention, the BP and GH in SF-8 scores were significantly improved by MA product consumption (45.9 ± 8.4 to 48.3 ± 7.5 and 45.6 ± 6.2 to 49.3 ± 4.7, *p* = 0.03 and 0.02, respectively) (Fig. 5C and D). In addition, at week 16, VT score was improved by MA product consumption compared to baseline (47.9 ± 5.1 to 50 ± 4.7, *p* = 0.09) (Fig. 5E). Furthermore, at week 8, compared to the baseline, the scores for RP in SF-8 and PF were improved by MA product consumption (45.7 ± 8.0 to 48 ± 5.5 and 44 ± 8.2 to 46.4 ± 5.8, *p* = 0.03 and 0.05, respectively) (Fig. 5A and B). The mean baseline score of each domain of the SF-8 of subjects and at 16 weeks is illustrated as a reader chart in Fig. 6, showing the progressive increase in the score of all domains after MA product consumption. In particular, the physical component of SF-8, BP, and GH were influenced by MA product consumption (Fig. 6). Furthermore, the mean PCS score was significantly improved from baseline to 8, 12, and 16 weeks after MA product consumption (43 ± 9.4 to 46.5 ± 6.2, 46.5 ± 5.5 and 45.4 ± 5.2, *p*<0.01, *p*<0.01 and *p* = 0.03, respectively), but not MCS, as shown in Fig. 7.

The secondary endpoints are body composition such as body weight and BMI. The change in body weight and BMI mean values of both groups before and after supplementation was not significant but tend to decrease after MA jelly consumption (58.2 ± 13.1 to 57.9 ± 12.9 and 24.2 ± 4.9 to 23.9 ± 4.3, *p* = 0.34 and 0.23, respectively) (data not shown).

### Stratified analysis by location

In order to verify the regional influence, such as differences of the age and the customs specific to Miyano and Oura area, we performed a stratified analysis zone-by-zone. The zone-by-zone data on the participants’ baseline and week 16 are shown in Table 3. At Miyano area, VAS score was significantly decreased at 16 weeks of MA product consumption compared to the baseline (–12.8 mm, *p* = 0.02). Furthermore, at week 16, PF (+3.3 point, *p* = 0.03), VT (+4.8 point, *p* = 0.03), RE (+4.8 point, *p* = 0.02) and PCS (+3.6 point, *p* = 0.04) were significantly improved while that of BP (+4.6 point, *p* = 0.08) also had the same tendency to improve after consumption of the MA product compared to baseline (Table 3). Furthermore, body weight and BMI values were significant decreased after MA product consumption (–0.95 kg, *p*<0.01 and –0.41 kg/m^2^, *p*<0.01, respectively). On the other hand, at Oura area, VAS score, JKOM score, and eight health domain of SF-8 were not significant changed after MA product consumption (Table 3).

### Adverse events

No serious, adverse reaction from taking MA jelly products was observed in this clinical study.

## Discussion

This is the first community resident-based clinical study that examined the effect of intake of maslinic acid (MA) in jelly product on the elderly suffering from chronic knee pain residing in an isolated island.

In the primary endpoints, participants who consumed MA jelly exhibited significant improvement in their JKOM score at week 8 compared to the baseline (Fig. 4). In addition, VAS showed a tendency to decrease as shown by the lower VAS scores at 4 and 16 weeks compared to baseline of MA jelly consumption (Fig. 3). Furthermore, the data obtained showed that MA product consumption significantly improved the score of BP, GH, and the PCS of the domain of SF-8 between baseline and week 16 (Fig. 6 and 7). The mean score of each domain of the SF-8 of subjects at baseline and 16 weeks is shown in Fig 6, wherein a progressive increase on the score of all domains, PF, RP, BP, GH, VT, SF, RE and MH, after MA products consumption for 16 weeks were observed. Our previous study has reported that MA in olive fruit is likely to improve joint pain and physical components of QOL not mental, by having an anti-inflammation effects, as well as reduction in the pain and QOL burden in humans with mild knee joint pain.^(8,11)^ Furthermore, Yap *et al.*^(16)^ has reported that the anti-inflammatory effects of pentacyclic triterpenoids including MA are largely ascribed to their ability to inhibit molecular targets such as 5-lipoxygenase, inducible nitric oxide synthase, cyclooxygenase 2, and nuclear factor-kappa B activities. Based on these findings, consumption of MA-rich olive fruit will likely improve physical-related QOL more than its effect on mental-related QOL.

In the present study, we performed a stratified analysis to take account of the difference in regional influences such as the age and the habit or customs of the subjects (Table 2 and 3). At Miyano area, knee joint pain (VAS and JKOM score) and health-related QOL (SF-8) were significantly improved by MA product consumption at 16 weeks compared to the baseline (Table 3). On the other hand, at Oura area, there was no significant improvement in any of the parameters at week 16 compared to the baseline (Table 3). In this study, the age of the subjects from Miyano was significantly higher than the age of subjects from Oura (73.6 ± 13.6 vs 68.6 ± 6.3, *p* = 0.01) (Table 2). In addition, at Miyano area, the knee joint pain (VAS and JKOM score), and QOL (SF-8) baseline scores were generally higher than those of Oura area (Table 2). From these results, we considered that the effectiveness of MA in olive product was effective on the group of subjects from Miyano area based on the baseline of physical-related QOL and pain more than on the subjects from Oura, although the scores of Oura were also slightly improved if we compare the scores at baseline and at the end of the study (Table 3). Therefore, we can also consider that the subjects group from Miyano is more responsive to the effect of MA on alleviating pain and improvement of QOL than the subjects from Oura area (Table 3).

Furthermore, we can see that based on the stratified analysis, the body weight and BMI values of subjects from Miyano area alone were significantly decreased after MA products consumption although the mean of those values tend to decrease if we compare the mean values before and after MA products consumption as a whole (Table 3). Based on this result, we can also deduce that MA intake has an anti-obesity effect that likely reduces the weight burden on the knees. These results are in agreement with the findings of our previous preliminary study wherein intake of 500 mg olive fruit extract/day (50 mg as MA) exhibited significant decrease in body weight and BMI, as well as improvement of mild knee joint pain, and for middle-aged and elderly healthy individuals experiencing or suffering from knee pain when climbing stairs, in randomized, double-blind, placebo-controlled trial.^(11)^ Liu *et al.*^(17)^ has also reported that MA normalized the depressed plasma adiponectin levels for anti-obesity hormone, increased epididymal fat pads weight, and insulin resistance in KK-Ay mice known as obesity model. Furthermore, since the body weight and BMI baseline values were significant higher at Miyano area compared to Oura area, these values at only Miyano were easy to decrease, but not Oura. Therefore, MA products intake improved joint pain and QOL by promoting weight loss, leading to reduce the knee burden in a group of elderly individuals.

One of the features of this present study, conducted as a “the community residents-based clinical study”, is the examination of the effect of intake of MA in jelly product on the elderly with chronic knee pain in general. Although it is important to verify the effects of the clinical trial which focuses on the selected subjects that excluded those with severe knee osteoarthritis and osteoarthritis with trauma, germ or gout as our previous clinical study,^(11)^ in this study, the recruited participants were chosen following a selection criteria of “general inhabitants with chronic knee pain” residing in an isolated island. This present study has an advantage with respect to finding out the beneficial effects of MA product in this community resident-based clinical study.

However, this community resident-based study has a relatively small number of subjects and was conducted as an open-label study, so no placebo control was used. The verification of the beneficial effects of this study, and obtaining of the data on MA product intake will require a larger cohort study and need to be confirmed in well-controlled trials. For example, we would like to pursue the goal of Nagata *et al.*^(18)^ report for large number of community-based study, which was established in September 1992 with residents in Takayama, Japan, indicating an inverse association of soy product intake with hot flashes. Furthermore, for its close study the protective effect of MA against chronic knee pain, future studies will be needed to analyze biochemical markers of cartilage turnover [*N*-propeptide of type IIA procollagen (PIIANP) and collagen type-II cleavage (C2C)] and inflammation marker [C-reactive protein (CRP)].^(19–21)^

In conclusion, we investigated the effects of MA containing product (30 mg MA), using a practical method to facilitate intake on a daily basis, on 29 elderly residents (mean 70.7 ± 10.1 years) of Nakajima Island (Ehime, Japan) populated by elderly individuals or “aging island” and reflect the aging rate of Japan in the next 50 years. Our results indicated that MA in olive fruit is likely to improve physical-related QOL more than mental-related QOL in the elderly community residents suffering from pain making QOL burden higher. Therefore, long-term daily use of MA products such as olive fruit may be useful for maintaining good health, health care, fall-prevention, and QOL improvement for the elderly in the aging Japanese society in the future.

## Figures and Tables

**Table 1:** Composition of maslinic acid jelly content*

Olive fruit extract (mg)	300.0
Calcium stearate (mg)	60.0
Gelling agent (mg)**	120.0
Edulcorant (mg)***	18.0
Emulsifying agent (mg)	10.0
Aroma chemical (mg)	30.0
Grapefruitseed extract (mg)	30.0
Water (mg)	9,432.0

*Values per 10 g of the test jelly. **Polysaccharide thickener. ***Sucralose.

**Table 2:** Baseline characteristics of the study subjects*

Characteristics	Miyano area (*n* = 13)	Oura area (*n* = 16)	Total (*n* = 29)	*p* value (Miyano vs Oura)
Age (years)	73.6 ± 13.6	68.4 ± 6.3	70.7 ± 10.1	**0.01**
Gender (number of participants)				
Male	5	1	6	0.06
Female	8	15	23	
Body composition				
Body weight (kg)	60.2 ± 17.5	56.6 ± 8.2	58.2 ± 13.1	0.91
BMI (kg/m^2^)	24.9 ± 6.0	23.6 ± 3.9	24.2 ± 4.9	0.52
JKOM				
VAS (mm)	41.3 ± 19.0	19.2 ± 28.6	29.1 ± 26.8	**<0.01**
JKOM score	28.5 ± 13.6	18.6 ± 21.1	23.1 ± 18.5	**<0.01**
SF-8 (points)				
Physical functioning	41.6 ± 4.3	46.0 ± 10.1	44.0 ± 8.2	**0.01**
Role physical	44.1 ± 5.0	47.0 ± 9.7	45.7 ± 8.0	0.07
Bodily pain	42.5 ± 8.2	48.7 ± 7.8	45.9 ± 8.4	**0.04**
General health	44.2 ± 5.7	46.7 ± 6.5	45.6 ± 6.2	0.21
Vitality	46.3 ± 3.9	49.2 ± 5.7	47.9 ± 5.1	0.16
Social functioning	43.8 ± 6.9	46.2 ± 9.1	45.1 ± 8.1	0.34
Role emotional	43.7 ± 5.9	49.7 ± 5.8	47.0 ± 6.5	**<0.01**
Mental health	46.6 ± 5.2	51.2 ± 6.3	49.1 ± 6.2	**0.03**
PCS	41.1 ± 6.3	44.5 ± 11.3	43.0 ± 9.4	**0.03**
MCS	46.3 ± 6.9	50.3 ± 7.0	48.5 ± 7.1	0.08

*Values are expressed as means ± standard deviation. BMI, body mass index; JKOM, Japanese Knee Osteoarthritis Measure; SF, Short Form; PCS, physical component summary; MCS, mental component summary.

**Table 3:** Effects of daily ingestion of maslinic acid on the physical and mental QOL and body composition of the subjects in Miyano and Oura area*

	Miyano (*n* = 13)			Oura (*n* = 16)	
	0 week**	16 week		0 week**	16 week
JKOM					
VAS (mm)	41.3 ± 19.0	28.5 ± 17.0*		19.2 ± 28.6	17.7 ± 25.3
JKOM score	28.5 ± 13.6	24.0 ± 12.4		18.6 ± 21.1	16.8 ± 15.0
SF-8 (points)					
Physical functioning	41.6 ± 4.3	45.0 ± 4.7*		46.0 ± 10.1	46.3 ± 5.9
Role physical	44.1 ± 5.0	46.6 ± 4.3		47.0 ± 9.7	47.1 ± 7.2
Bodily pain	42.5 ± 8.2	47.1 ± 6.6^†^		48.7 ± 7.8	49.3 ± 8.3
General health	44.2 ± 5.7	47.8 ± 4.6		46.7 ± 6.5	50.5 ± 4.6
Vitality	46.3 ± 3.9	50.9 ± 4.7*		49.2 ± 5.7	49.3 ± 4.7
Social functioning	43.8 ± 6.9	45.4 ± 7.8		46.2 ± 9.1	46.1 ± 9.6
Role emotional	43.7 ± 5.9	48.5 ± 3.3*		49.7 ± 5.8	49.8 ± 6.1
Mental health	46.6 ± 5.2	49.0 ± 6.9		51.2 ± 6.3	50.2 ± 8.0
PCS	41.1 ± 6.3	44.6 ± 5.2*		44.5 ± 11.3	46.0 ± 5.3
MCS	46.3 ± 6.9	49.1 ± 5.5		50.3 ± 7.0	49.5 ± 6.8
Body composition					
Body weight (kg)	60.2 ± 17.5	59.3 ± 17.4**		56.6 ± 8.2	56.7 ± 8.2
BMI (kg/m^2^)	24.9 ± 6.0	24.5 ± 5.9**		23.6 ± 3.9	23.4 ± 2.6

*Mean values with their standard deviation, ^†^*p*<0.1, *p<0.05, **p<0.01 vs 0 week (baseline). **Baseline data. BMI, body mass index; JKOM, Japanese Knee Osteoarthritis Measure; SF, Short Form; PCS, physical component summary; MCS, mental component summary.

## Abbreviations

BMI: body mass index
BP: bodily pain
C2C: collagen type-II cleavage
GH: general health
JKOM: Japanese Knee Osteoarthritis Measure
JOA: The Japanese Orthopaedic Association
MA: maslinic acid
MCS: mental component summary
MH: mental health
PCS: physical component summary
PF: physical functioning
PIIANP: *N*-propeptide of type IIA procollagen
QOL: quality of life
RE: role emotional
RP: role physical
SF: social functioning
SF-8: Short Form 8 Healthy Survey
VAS: visual analogue scale
VT: vitality
